# Apoptosis Induction by MEK Inhibition in Human Lung Cancer Cells Is Mediated by Bim

**DOI:** 10.1371/journal.pone.0013026

**Published:** 2010-09-27

**Authors:** Jieru Meng, Bingliang Fang, Yong Liao, Christine M. Chresta, Paul D. Smith, Jack A. Roth

**Affiliations:** 1 Department of Thoracic and Cardiovascular Surgery, The University of Texas M. D. Anderson Cancer Center, Houston, Texas, United States of America; 2 Department of Experimental Therapeutics, The University of Texas M. D. Anderson Cancer Center, Houston, Texas, United States of America; 3 Cancer and Infection Research, Astrazeneca, Macclesfield, United Kingdom; Wayne State University, United States of America

## Abstract

AZD6244 (ARRY-142886) is an inhibitor of MEK1/2 and can inhibit cell proliferation or induce apoptosis in a cell-type dependent manner. The precise molecular mechanism of AZD6244-induced apoptosis is not clear. To investigate mechanisms of AZD6244 induced apoptosis in human lung cancer, we determined the molecular changes of two subgroups of human lung cancer cell lines that are either sensitive or resistant to AZD6244 treatment. We found that AZD6244 elicited a large increase of Bim proteins and a smaller increase of PUMA and NOXA proteins, and induced cell death in sensitive lung cancer cell lines, but had no effect on other Bcl-2 related proteins in those cell lines. Knockdown of Bim by siRNA greatly increased the IC_50_ and reduced apoptosis for AZD6244 treated cells. We also found that levels of endogenous p-Thr32-FOXO3a and p-Ser253-FOXO3a were lower in AZD6244-sensitive cells than in AZD6244-resistant cells. In the sensitive cells, AZD6244 induced FOXO3a nuclear translocation required for Bim activation. Moreover, the silencing of FOXO3a by siRNA abrogated AZD6244-induced cell apoptosis. In addition, we found that transfection of constitutively active AKT up-regulated p-Thr32-FOXO3a and p-Ser253-FOXO3a expression and inhibited AZD6244-induced Bim expression in sensitive cells. These results show that Bim plays an important role in AZD6244-induced apoptosis in lung cancer cells and that the PI3K/AKT/FOXO3a pathway is involved in Bim regulation and susceptibility of lung cancer cells to AZD6244. These results have implications in the development of strategies to overcome resistance to MEK inhibitors.

## Introduction

Activation of the Ras/Raf/MEK/MAP kinase pathway has been implicated in uncontrolled cell proliferation and tumor growth. AZD6244 (ARRY-142886), a novel, selective, ATP-uncompetitive inhibitor of mitogen-activated protein kinase kinase 1/2 (MEK1/2), has shown activity in nanomolar concentrations against isolated MEK enzyme and numerous cancer cell lines [1]. In vitro studies showed that AZD6244 down-regulated levels of p-ERK efficiently. AZD6244 has shown activity in several tumor xenograft models of human cancer [2]–[4]. In clinical trials, whilst patients from several tumor types have shown responses to MEK inhibitor monotherapy, other patients' tumors, particularly non-small cell lung cancers, are inherently resistant to MEK inhibition. Therefore it is important to understand the underlying mechanisms responsible for resistance to MEK inhibition in the event it becomes important therapeutic modality in this very common cancer.

Our previous study [5] showed that the MEK inhibitor AZD6244 potently inhibited proliferation at nanomolar concentrations in Calu-6, H2347, and H3122 lung cancer cell lines but had little effect on H196, Calu-3, H522, or HCC2450 cell lines. In addition, we found that following sub-G1 cell cycle arrest, 20–40% of AZD6244-sensitive cells underwent apoptosis, we observed no apoptosis in AZD6244-resistant cells. We previously showed that p-AKT expression is low in AZD6244-sensitive lung cancer cell lines but high in resistant cells, suggesting that p-AKT is a mediator of resistance to AZD6244 treatment. In this paper we investigate downstream mediators in AZD6244-induced apoptosis in human lung cancer cells.

Apoptosis could be regulated via extrinsic (death receptor) or intrinsic (mitochondrial) cell death pathways. Intrinsic apoptosis is mediated by the Bcl-2 family proteins, consisting of three subfamilies: the pro-survival members, such as Bcl-2 or Mcl-1, the pro-apoptotic Bax/Bak subgroup, and the pro-apoptotic Bcl-2 homology 3-only (BH3-only) proteins. Apoptotic stimuli trigger activation of specific BH3-only proteins, which then engage the pro-survival Bcl-2 family members and liberate the downstream effectors, Bax and Bak, to elicit mitochondrial outer membrane permeabilization, unleashing the caspase cascade and culminating in cell death. Bim, p53-up-regulated modulator of apoptosis (PUMA) and NOXA have been recently reported to play an important role in chemotherapy and targeted therapy induced apoptosis in breast cancer [6], leukemia [7], myeloma [8] and NSCLC [9] cells.

The FOXO transcription factor members promote or inactivate multiple target genes involved in tumor suppression, such as *Bim*, *FasL*, and *TRAIL* genes for inducing apoptosis [10], [11], *p27kip1*, *cyclin D15* for cell cycle regulation [12], and *GADD45a* for DNA damage repair [13]. FOXO3a is one of the most important FOXO family of transcription factors that have a wide range of cellular functions. FOXO3a are phosphorylated and inactivated by AKT through phosphorylation at Thr32, Ser253, and Ser315 which results in nuclear export and inhibition of its transcription activity [14], [15]. FOXO3a has also been shown to be regulated by the oncoprotein ERK [16] at three ERK phosphorylation sites, Ser 294, Ser 344, and Ser 425. As with AKT, phosphorylation of these serine residues with ERK increased FOXO3a cytoplasmic distribution and nuclear export.

Because the balance between antiapoptotic and proapoptotic proteins is critical to drug-induced apoptosis, we evaluated changes in Bcl-2 family proteins in AZD6244 sensitive and resistant lung cancer cell lines and found that the MEK inhibitor AZD6244 up-regulates the proapoptotic BH3-only proteins Bim, PUMA and NOXA, a process associated with subsequent cell death. We also found that silencing either FOXO3a, a transcriptional regulator of Bim, or Bim with small interfering RNA (siRNA) greatly inhibited apoptosis. Furthermore, expression of constitutively active AKT (caAKT) in sensitive cells inhibited AZD6244-induced Bim over-expression and led to AZD6244 resistance. In contrast, stable transfection of dominant-negative AKT into resistant cells enhanced AZD6244-induced Bim over-exrpession.

## Materials and Methods

### Materials

AZD6244, provided by AstraZeneca Pharmaceuticals (Macclesfield, UK), was dissolved to 25 mM in dimethyl sulfoxide (DMSO) and stored at –80°C. Antibody against Bim was purchased from Calbiochem (San Diego, CA). Antibodies against p-ERK, FOXO3a, p-FOXO3a (Thr32), p-FOXO3a (Ser253), Bad, PARP, NOXA and PUMA, and the AKT kinase assay kit were purchased from Cell Signaling Technology (Danvers, MA). Antibodies against Bak, Bcl-xl and Caspase-9 were purchased from Santa Cruz Biotechnology (Santa Cruz, CA). Predesigned FOXO3a siRNA were purchased from Santa Cruz Biotechnology (Santa Cruz, CA), and Bim and control siRNA were from Qiagene (Valencia, CA)**.** The full-length human BimEL cDNA, which is cloned into the expression vector pCMV6-XL4, was purchased from OriGene Technologies (Rockville, MD). Protease inhibitor cocktail, β-actin antibody, and sulforhodamine B (SRB) were from Sigma Chemical Corporation (St. Louis, MO). Protein assay materials and SYBR Green Supermix were purchased from Bio-Rad **Laboratories** (Hercules, CA), and Geneticin was from Life Technologies Corporation (Carlsbad, CA). Lipofactamin 2000 and Trizol reagent were purchased from Invitrogen Corporation (Carlsbad, CA), and reverse transcription reagents were from Applied Biosystems Inc. (Foster City, CA). DeadEnd™ Flurometic TUNEL System was purchased from Promega (Madison, WI).

### Cell culture

Cell lines H2347, H3122, H196, HCC2450, and H522 were provided by Drs. A. Gazdar and J. Minna, Hamon Center for Therapeutic Oncology Research, The University of Texas Southwestern Medical Center, Dallas, TX. All lung cancer cell lines were maintained in high-glucose Dulbecco's modified Eagle's medium (DMEM) supplemented with 10% fetal bovine serum (FBS), 100 µg/mL ampicillin, and 0.1 mg/mL streptomycin; the cells were cultured at 37°C in a humidified atmosphere containing 5% CO_2_ and 95% air.

### Cell viability assay

Cell viability was determined by using the SRB assay, and each assay was carried out in quadruplicate. Lung cancer cells were seeded at about 3,000 per well in 96-well plates and incubated for 24 hours in DMEM supplemented with 10% FBS. The cells were then treated with AZD6244 at the indicated concentrations which were equivalent to serum concentrations achieved in patients after oral administration. Cells treated with DMSO were used as controls. Cells were fixed 96 hours after treatment by adding 50 µL of 10% trichloroacetic acid at 4°C for 1 hour. They were then stained with 70 µL of 0.4% SRB for 60 minutes and washed with 1% acetic acid; 200 µL of Tris base (10 mmol/L; pH, 10.5) was added. Absorbance readings at 570 nm were determined by using a microplate analyzer. The relative survival rate (%) was calculated by the equation OD_T_/OD_C_ × 100% (with OD_T_ representing the absorbance of treatment groups, and OD_C_ the absorbance of control groups). Median inhibitory concentrations (IC_50_ values) were determined by using CurveExpert 1.3 software and plotted in dose-response curves. Experiments were repeated at least three times.

### Western blot analysis

Whole-cell lysates were prepared by washing the cells with phosphate-buffered saline (PBS) and subjecting them to lysis with Laemmli sample buffer supplemented with protease inhibitor cocktail. After the lysates were sonicated for 15 seconds, the protein concentrations were quantified by using the Bio-Rad protein assay kit. Equivalent proteins were loaded, separated by 10% or 12% sodium dodecyl sulfate–polyacrylamide (SDS-PAGE) gel electrophoresis, and then transferred to nitrocellulose membranes at 80 V for 2 hours. The membranes were blocked for 1 hour with 5% nonfat dried milk in Tris buffer containing 0.1% Tween (TBST) and probed with diluted primary antibody at 4°C overnight. The membranes were then washed three times in TBST buffer and probed with infrared dye–labeled secondary antibodies; the immunoreactive bands were visualized with use of the Odyssey® Imager (Li-COR Biosciences, Lincoln, NE).

### Cell cycle and apoptosis assay

Cells were harvested by trypsinization, washed twice in cold PBS, fixed with ice-cold 70% methanol, and incubated at 4°C overnight. Cells were then washed with PBS and incubated with 25 µg/mL propidium iodide containing 30 µg/mL ribonuclease for 30 minutes at room temperature. Cells were analyzed on an EPICS Profile II flow cytometer (Coulter Corp., Hialeah, FL) with the Multicycle Phoenix Flow Systems program (Phoenix Flow Systems, San Diego, CA). Experiments were repeated at least three times.

### Measurement of apoptosis by TUNEL (terminal deoxynucleotidyl transferase mediated nick-end labeling) assay

The TUNEL assay was performed following the instructions provided by the manufacturer of a commercially available kit (DeadEnd™ Fluorometric TUNEL System) from Promega. Apoptotic cells exhibit a strong nuclear green fluorescence that could be detected using a standard fluorescein filter. All cells stained with DAPI exhibit a strong blue nuclear fluorescence. The slides were observed under fluorescence microscopy with relative apoptotic cells determined by counting TUNEL-positive cells in five random fields (at ×100 magnification) for each sample.

### Real-time PCR

Total RNA was isolated by using Trizol reagent and reverse transcribed to cDNA. As previously described, we used Bim primers [6] in our study. Quantitative polymerase chain reaction (PCR) was performed in 25 µL of mixture, with 12.5 µL of 2× SYBR Green Supermix, 1 µM of each forward and reverse primer, and 4 to 12 ng of template, using the CFX96 real-time PCR detection system (Bio-Rad). PCR was performed for an initial denaturation of 10 minutes at 95°C followed by 39 cycles of 15 seconds at 95°C, 30 seconds at 58°C, and 30 seconds at 72°C. All samples were analyzed in triplicates, and human glyceraldehyde 3-phosphate dehydrogenase (GAPDH) was used as an endogenous control. Relative expression was calculated by using the 2–δδCt method.

### siRNA and Bim cDNA transfection

Cells were cultured in 6-well plates until 70% confluent and transfected with 200 nmol/L of control nonspecific siRNA, Bim-targeted siRNA, or FOXO3a-targeted siRNA by using Lipofectamine™ 2000 according to the manufacturer's instructions. Twenty-four hours after transfection, the cells were treated with DMSO (control) or AZD6244 at indicated doses and time points. The cells were then collected and processed for immunoblotting or propidium iodide staining for the cell cycle assay.

For Bim cDNA transfection, cells were also cultured in 6-well plates until 70% confluent and transfected with control vector or BimEL expression vector, at a concentration of 4 µg in 250 µl medium, using Lipofectamine 2000. Forty-eight hours after transfection, the cells were harvested for immunoblotting or fixed with 4% formaldehyde for TUNEL assay.

### AKT kinase activity assay

Cell were washed twice with PBS, subjected to lysis in cell lysis buffer, and sonicated for 15 seconds. The extracts were centrifuged to remove cellular debris, and the protein concentrations of the supernatants were determined by using Bio-Rad protein assay reagent. A 200- µL cell lysate sample was incubated with 20 µL of immobilized anti-AKT antibody at 4°C overnight with gentle rocking. The resulting immunoprecipitates were washed three times with lysis buffer and twice with AKT kinase buffer. Kinase assays were performed for 30 minutes at 30°C under continuous agitation in kinase buffer containing 200 µM ATP and 1 µg of GSK-3 fusion protein. Reaction products were resolved by 10% SDS-PGAE, followed by Western blotting with an anti-phospho-GSK-3α/β antibody according to the manufacturer's instructions for the nonradioactive AKT kinase assay. Experiments were repeated at least three times.

### Immunofluorescence staining

Cells were cultured on CultureSlides (BD Biosciences, CA). The medium was aspirated, and the cells were washed three times with PBS and then fixed with freshly prepared 4% paraformaldehyde for 30 minutes at room temperature. After another washing step with PBS, cells were permeabilized for 20 minutes at room temperature by using PBS buffer containing 0.2% Triton X-100 and 0.1% sodium citrate. Then the cells were incubated in PBS containing 5% nonfat dry milk at room temperature for 1 hour. Primary antibody incubation was carried out with anti-FOXO3a (1∶100 dilutions) at 4°C overnight. After another washing step with PBS, the cells were incubated with the secondary antibody, FITC-conjugated anti-rabbit antibody (1∶100; Jackson ImmunoResearch Laboratories, Inc., West Grove, PA) for 30 minutes at room temperature. All antibodies were diluted in PBS plus 5% nonfat dry milk. The slides were then stained with Prolong antifade solution (Molecular Probes, Inc., Eugene, OR) for 5 minutes at room temperature followed by washing three times in PBS. Images were acquired by fluorescence microscopy with an inverted Zeiss laser-scanning microscope. Individual nuclei were outlined by using DAPI fluorescence, and the nuclear fluorescence of Cy3 was quantified by using Zeiss KS400 image analysis software (Carl Zeiss, Inc., Oberkochen, Germany). Experiments were repeated at least three times.

### Statistical analysis

Data were expressed as the mean ± SD and calculated as the mean values with 95% confidence intervals. Statistical comparison between experimental groups was performed by two-way ANOVA test by using Microsoft Excel software. Values of *P*<0.05 were considered statistically significant.

## Results

### AZD6244 increases Bim expression in lung cancer cell lines

Our previous study [5] showed that the AZD6244 inhibited proliferation in Calu-6, H2347, and H3122 lung cancer cell lines but had little effect on H196, Calu-3, H522, or HCC2450 cell lines. In addition, we found that following sub-G_1_ cell cycle arrest, 20–40% of AZD6244-sensitive cells underwent apoptosis, but we observed no apoptosis in AZD6244-resistant cells. In this study, we used these same cell lines to further determine the mechanisms of AZD6244-induced apoptosis.

**Figure 1:**
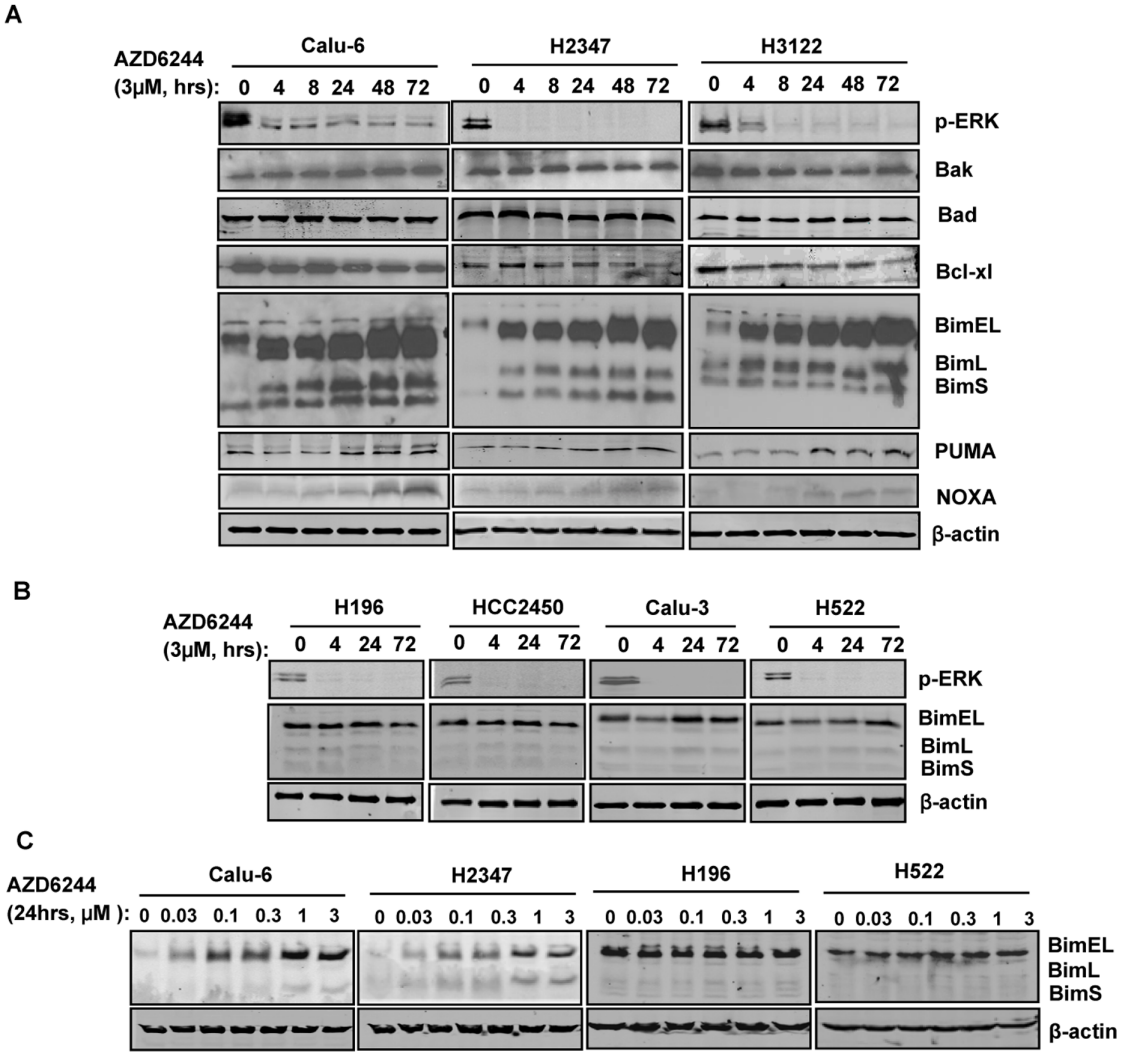
The expression of various Bcl-2 family proteins in lung cancer cells after AZD6244 treatment. Western blots of Bcl-2 family members after treatment with AZD6244. (A) Human lung cancer cell lines (Calu-6, H2347, and H3122) were treated with 3 µM AZD6244 for 4, 8, 24, 48, and 72 hours. (B) Human lung cancer cell lines (Calu-3, H196, H522, and HCC2450) were treated with 3 µM AZD6244 for 4, 24, and 72 hours. (C) Calu-6, H2347, H196 and H522 cells were treated with 0.03, 0.1, 0.3, 1 and 3 µM of AZD6244 for 24 hours. Data represent one of three independent experiments with similar results.

The mitochondrial apoptotic pathway is known to play a critical role in tyrosine kinase inhibitor–induced apoptosis [6]–[9]. To evaluate which Bcl-2 family members are critically affected by AZD6244 treatment, we determined their protein levels in the three sensitive lung cancer cell lines after treatment with 3 µM AZD6244, the concentration reached in the serum of patients receiving oral AZD6244. Calu-6 has a mutant KRAS and wildtype BRAF while H2347 in mutant NRAS and H3122 [17] have both wildtype KRAS and BRAF. Western blot analysis showed that treatment with AZD6244 induced rapid and sustained increases in levels of BimEL and, to a lesser extent, of BimL and BimS, in all sensitive cell lines (Fig. 1A). Furthermore, treatment with sub-micromolar concentrations (0.03, 0.1, 0.3, 1, and 3 µM) of AZD6244 for 24 hrs induced marked increase in levels of Bim (Fig. 1C). These findings indicated that AZD6244 induced its effects on Bim expression in a concentration- and time-dependent manner. However, in these cells, the levels of other Bcl-2 family members (Bax, Bak, and Bcl-xL) did not change noticeably at any concentration of AZD6244 or at any time point (Fig. 1A). In contrast, AZD6244 did not induce obvious changes in Bim expression in resistant cell lines (Fig. 1B). The resistant cell lines are all wild type for BRAF and KRAS. We also detected suppression of p-ERK expression with AZD6244 in both sensitive and resistant cells (Fig. 1A and 1B). We also investigated the expression of BH3-only proteins PUMA and NOXA following 3 µM AZD6244 treatment. PUMA and NOXA expressions were increased upon AZD6244 treatment, however, the levels of the increase were much less than that observed with Bim (Fig. 1A). Since the upregulation of Bim is much more dramatic than PUMA and NOXA in AZD6244-treated cells, we focused on the role of Bim in subsequent studies.

### AZD6244-induced Bim overexpression is caused by both increased transcription and increased protein stability

**Figure 2:**
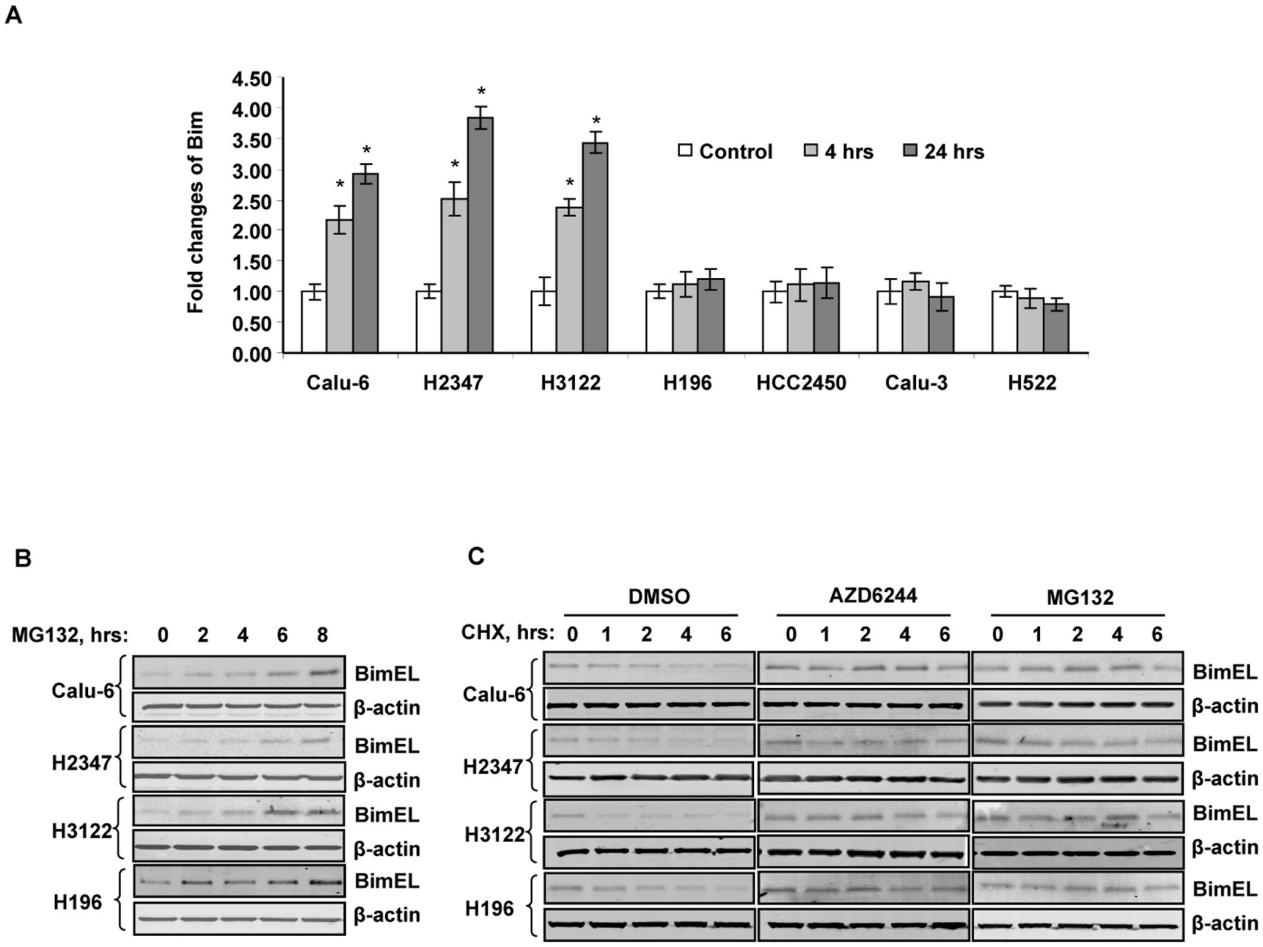
AZD6244 induced increase of *Bim* mRNA levels and Bim protein stabilization in lung cancer cells. (A) Total RNA was isolated in parallel. Expression of *Bim* was measured by real-time PCR, and normalized to the level of *GAPDH*. Data shown are representative of three independent experiments with similar results. *Columns*, mean; *bar*, SD. * , *P*<0.05, compared with untreated cells. (B) Calu-6, H2347, H3122 and H196 cells were treated with 30 µM MG132 for 2, 4, 6, and 8 hours and Western blot analysis with Bim expression was performed. (C) Calu-6, H2347, H3122 and H196 cells were treated with DMSO, 3 µM AZD6244, or 30 µM MG132 for 6 hours, and then with 25 µg/ml of cycloheximide to block protein synthesis. Western blot analysis with Bim expression was performed. Data represent one of three independent experiments with similar results.

To investigate the mechanisms of AZD6244-induced Bim expression, we analyzed the levels of Bim mRNA after treatment with AZD6244. Sensitive and resistant cells were treated with 3 µM AZD6244 for 4 and 24 hours, and cells were harvested for real-time PCR analysis. The mRNA levels of GAPDH were used as internal controls. The result showed that, after normalization with internal controls, treatment with AZD6244 led to a substantial increase in mRNA levels of Bim in a time-dependent manner in the sensitive Calu-6, H2347, and H3122 cells. AZD6244, at a concentration of 3 µM, increased Bim mRNA between 2.2- to 2.5-fold after 4 hrs, and 2.9- to 3.8-fold after 24 hrs incubation in these three cell lines (Fig. 2A). There are significant difference in Bim mRNA expression between treatment and control groups or between the 4 hrs and 24 hrs treatment groups (*P*<0.05 among all pairwise comparison). In contrast, AZD6244 could not induce Bim mRNA expression in the four resistant cell lines H196, HCC2450, Calu-3 and H522.

Because ERK1/2 signaling pathway activation phosphorylates Bim and promotes proteasome-dependent degradation of Bim [18], we tested whether Bim protein was stabilized by AZD6244 treatment. For this purpose, we first treated sensitive (Calu-6, H2347 and H3122) and resistant (H196) cells with proteosome inhibitor MG132 at 30 µM for 2, 4, 6 and 8 hours, harvested cells and detected Bim expression by Western blot (Fig. 2B). Bim protein levels increased 2 hours after exposure to MG132 and continued to increase at 6–8 hours. We then treated these cells with DMSO, 3 µM AZD6244, or 30 µM MG132 for 4 hours and then added 25 µg/ml cycloheximide to block protein synthesis in the cells. Cells were then harvested over time and Bim expression was detected by Western blot analysis (Fig. 2C). We found that Bim protein was rapidly degraded in cells treated with DMSO in all four tested cell lines. In contrast, in cells treated with AZD6244 or MG132, the Bim protein levels were stabilized even after 6 hours of cycloheximide treatment, indicating that degradation of Bim protein was blocked by treatment with AZD6244. Together, our results indicate that the increased BimEL expression induced by AZD6244 treatment could be caused by two mechanisms: an increase of *Bim* gene transcription and an inhibition of Bim protein degradation. As AZD6244 can inhibit Bim protein degradation in both sensitive and resistant cell lines, the increase in *Bim* gene transcription may be more significant in inducing AZD6244-induced apoptosis.

### Bim is required for AZD6244-induced apoptosis in lung cancer cells

**Figure 3:**
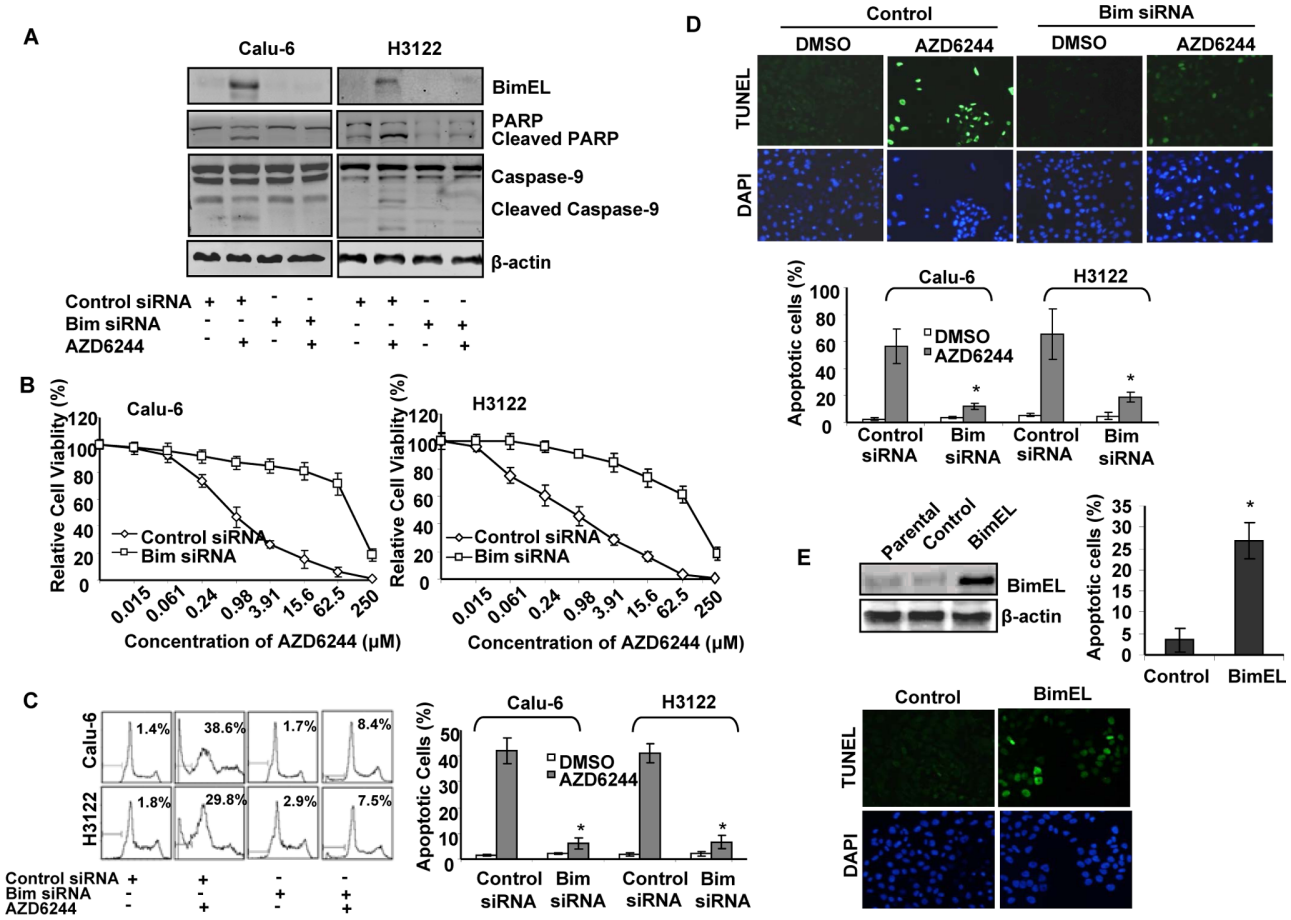
The effect of Bim-specific small interfering RNA (siRNA) on AZD6244-induced apoptosis. (A) Calu-6 and H3122 cells were transfected with Bim-specific or control siRNA and then treated with 3 µM AZD6244 for 48 hours. Expression of Bim, PARP and Caspase-9 were analyzed by Western blotting. (B) Cells were cultured in medium containing various concentrations of AZD6244 for 96 hours. Cell viability was determined by sulforhodamine B, and relative cell viability was plotted as described in the Materials and Methods section. Values represent mean ± SD of three independent triplicate assays. (C) Parallel cells were fixed with ethanol and stained with propidium iodide; DNA content was analyzed by flow cytometry. Numbers represent percentages of apoptotic sub-G_1_–phase cells. Data represent one of three independent experiments with similar results. *Columns*, mean; *bar*, SD. * , *P*<0.05, compared with the control siRNA transfected cells. (D) Parallel cells were also fixed for TUNEL and DAPI staining. Apoptotic cell nuclei in TUNEL staining were labeled with FITC and visualized under fluorescence microscopy. The relative apoptotic cells were determined by counting TUNEL positive cells in five random fields (at 100× magnification) for each sample. *Columns*, mean; *bar*, SD. *, *P*<0.05, compared with the control siRNA transfected cells. The representative photographs of Calu-6 were shown in the upper panel and the percentage of apoptotic cells of both Calu-6 and H3122 were showed in the lower panel. (E) Calu-6 cells were transfected with BimEL expression vector and control vector for 48 hrs. Expression of Bim was analyzed by Western blotting and apoptotic cells were detected with TUNEL assay.

To examine the role of Bim in AZD6244-induced cell apoptosis, we generated specific siRNA constructs for Bim in Calu-6 and H3122 cell lines. As shown in Fig. 3A, siRNA knockdown of Bim substantially inhibited the expression of Bim after treatment with 3 µM AZD6244 for 48 hours. PARP cleavage and caspase-9 cleavage/activation were inhibited.

We also tested the antiproliferative effect of AZD6244 on control and Bim siRNA–transfected cells by SRB assay and determined IC_50_ values. We found that the IC_50_ to AZD6244 increased from 0.7 to 76.3 µM in Calu-6 cells and from 1.4 to 89.3 µM in H3122 cells (Fig. 3B). The control and Bim siRNA–transfected cells were treated with 3 µM AZD6244 for 72 hours, and cells were harvested for cell cycle analysis. Results showed that after treatment with AZD6244, the percentage of apoptotic (sub-G_1_) cells decreased from 38.6% to 8.4% in Bim siRNA–transfected Calu-6 cells and from 29.8% to 7.5% in Bim siRNA–transfected H3122 cells (Fig. 3C). The TUNEL assay also indicated Bim siRNA transfection inhibited AZD6244-induced apoptosis, from 56.6% to 12.1% in Calu-6 and from 65.3% to 18.5% in H3122 cells respectively (Fig. 3D).

We further tested whether increased Bim expression is sufficient to induce apoptosis. A plasmid encoding full-length BimEL was transiently transfected to Calu-6 and apoptosis of the transfected cells was determined by TUNEL assay. We found that almost all cells transfected with control vector are TUNEL-negative after 48 hrs, whereas, BimEL expression vector transfected cells showed significantly increased TUNEL-positive apoptosis (Fig. 3E).

### The role of FOXO3a in AZD6244-induced Bim expression

**Figure 4:**
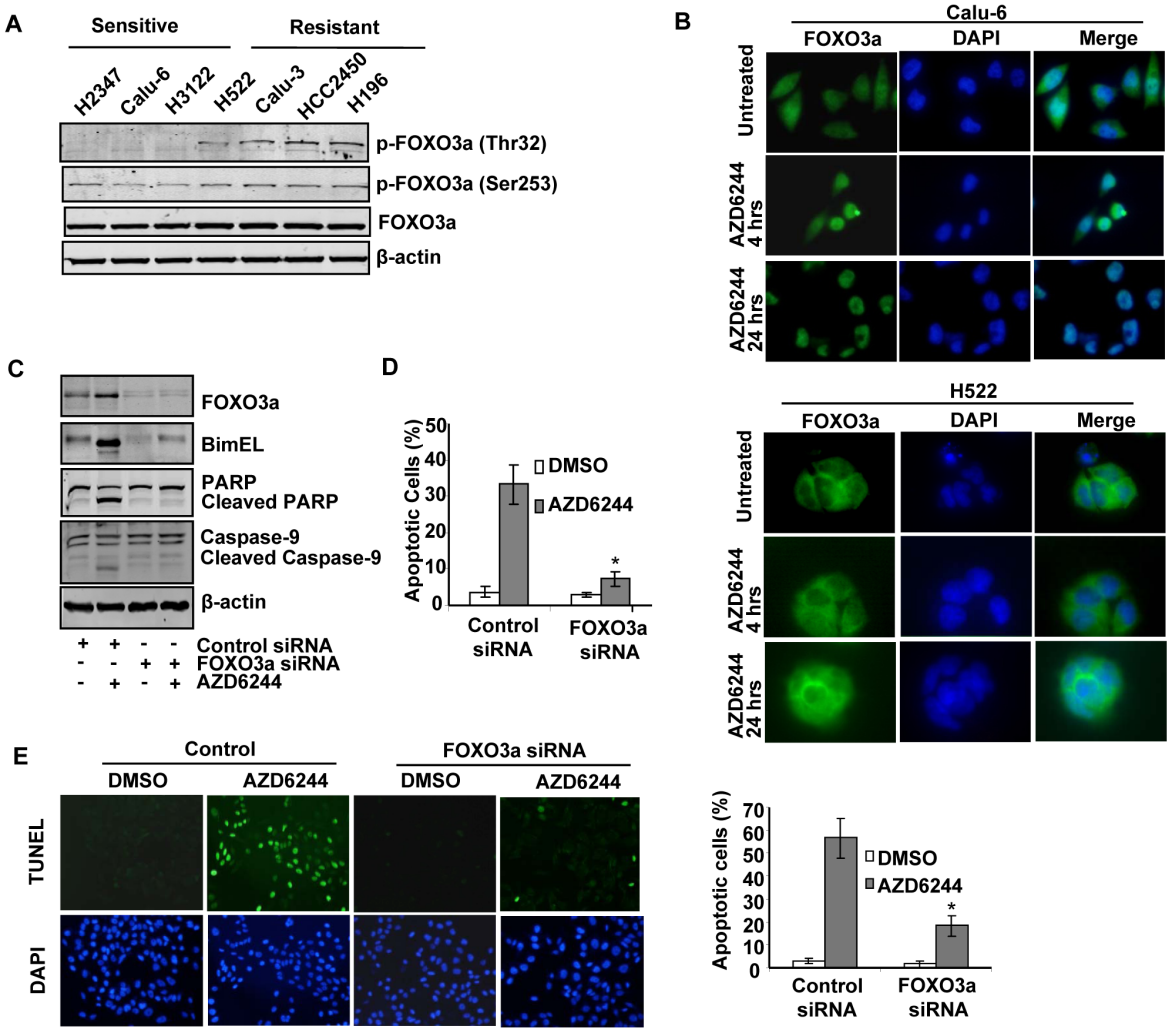
Direct role of FOXO3a in transcriptional regulation of Bim. (A) Endogenous expression of p-Thr32-FOXO3a, p-Ser253-FOXO3a, and total FOXO3a was detected in 8 cell lines. (B) Subcellular localization of FOXO3a in Calu-6 and H522 cells was detected with immunofluorescence staining after AZD6244 treatment. (C) Calu-6 cells were either mock-transfected or transfected with a FOXO3a-specific small interfering RNA (siRNA) and then treated with 3 µM AZD6244 for 4 hours. Expression of FOXO3a, Bim, PARP and Caspase-9 were analyzed by Western blotting. (D) In parallel, Calu-6 cells were fixed with ethanol and stained with propidium iodide; DNA content was analyzed by flow cytometry. Numbers represent percentages of apoptotic sub-G_1_–phase cells. Data represent one of three independent experiments with similar results. *Columns*, mean; *bar*, SD. * , *P*<0.05, compared with the control siRNA transfected cells. (E) TUNEL assays were performed as described in Fig. 3D. The representative photographs of Calu-6 are shown.

It has been reported that activation of FOXO transcription factors induces Bim mRNA expression and promotes cell apoptosis in a Bim-dependent manner [19], [20]. FOXO transcription factors are phosphorylated by AKT at three highly conserved sites, Thr32, Ser253, and Ser315, which leads to cytoplasmic retention and impairment of FOXO nuclear transcriptional activity. ERK also has been shown to phosphorylate FOXO3a and to increase its nuclear export. In our previous study [5], we found that p-AKT expression was much higher in resistant cells than in sensitive cells. As expected, endogenous levels of p-Thr32-FOXO3a and p-Ser253-FOXO3a were higher in resistant cells than in sensitive cells (Fig. 4A). No consistent differences were seen between the two groups for total FOXO3a.

Transcriptional activation of FOXO3a is highly influenced by its subcellular localization in a process tightly regulated by AKT and ERK. We investigated whether AZD6244 treatment caused FOXO3a to relocate to the nucleus where it is activated. Immunofluorescence staining showed that in sensitive Calu-6 cells, treatment with 3 µM AZD6244 induced considerable subcellular localization of FOXO3a from the cytoplasm to the nucleus. However, in resistant H522 cells, we detected no obvious changes in subcellular localization of FOXO3a after AZD6244 treatment. Moreover, we noticed that in untreated Calu-6 cells, FOXO3a resided in both the cytoplasm and nucleus; in untreated H522 cells, most of the FOXO3a resided in the cytoplasm, and nuclear staining was relatively negligible (Fig. 4B). These findings are consistent with those detected on Western blotting of p-FOXO3a expression (Fig. 4A).

To examine the role of FOXO3a in AZD6244-induced Bim, we evaluated the effect of specific siRNA constructs for FOXO3a in Calu-6 and H3122 cells. As shown in Fig. 4C, siRNA knockdown of FOXO3a inhibited the expression of FOXO3a, which resulted in strong suppression of AZD6244-induced Bim, PARP cleavage and caspase-9 cleavage/activation after treatment for 24 hours. Analysis of apoptosis following FOXO3a siRNA transfection in sensitive cells after AZD6244 treatment showed the percentage of sub-G_1_ apoptotic cells decreased from 32.5% to 10.9% after treatment with AZD6244 for 72 hours (Fig. 4D). The TUNEL assay also showed that FOXO3a siRNA transfection significantly inhibited AZD6244-induced apoptosis from 56.7% to 18.4% in Calu-6 (*P*<0.05, Fig. 4E). Our results suggested that FOXO3a activation is required for AZD6244-induced Bim expression.

### caAKT transfection up-regulates the expression of p-FOXO3a and inhibited AZD6244-induced apoptosis

**Figure 5:**
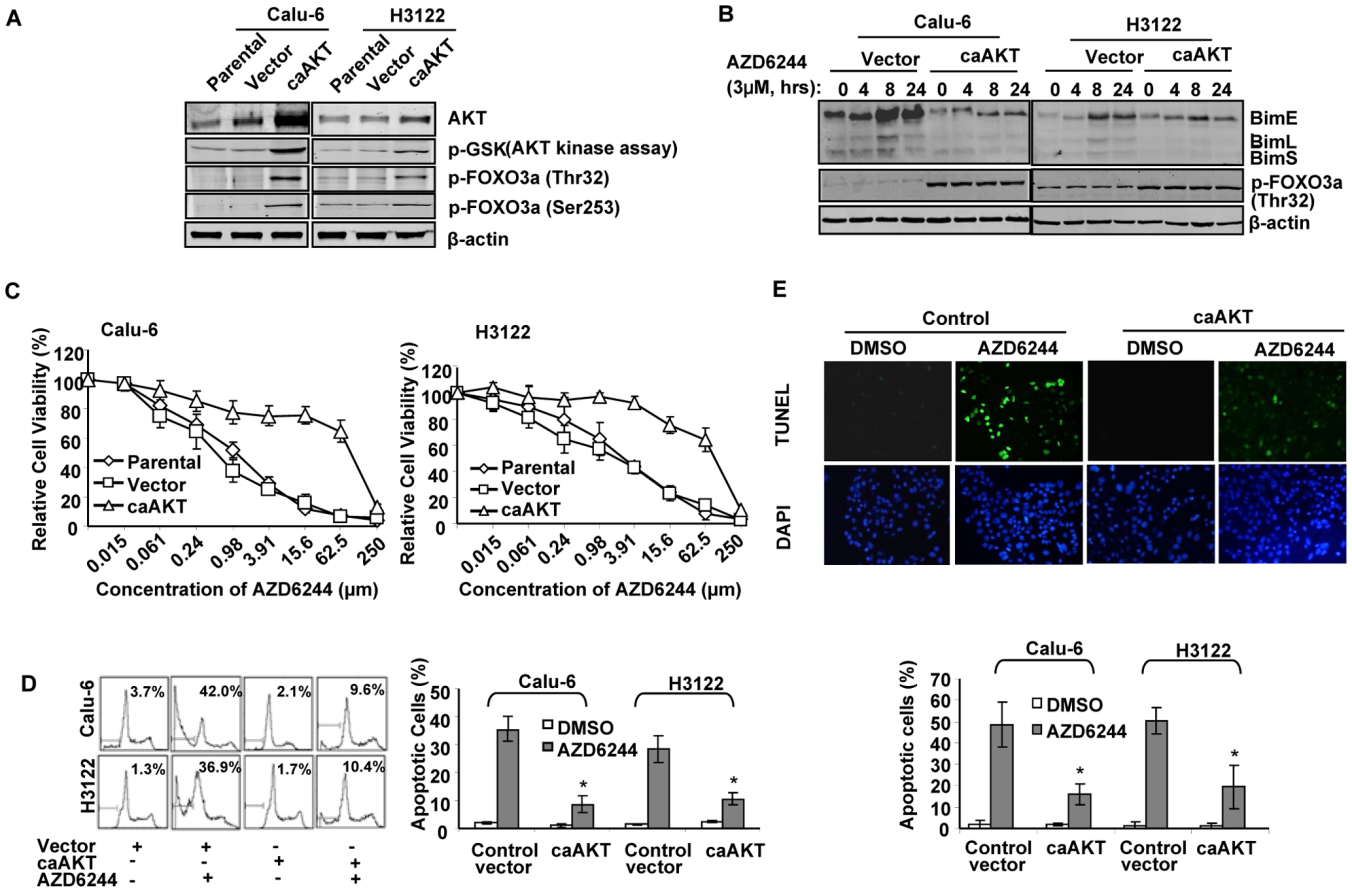
Effect of constitutively active AKT (caAKT) on FOXO3a-mediated Bim expression. (A) Lung cancer cell lines Calu-6 and H3122 were transfected with an empty retroviral vector or a caAKT-expressing vector. After a brief selection, AKT activity and p-Thr32-FOXO3a and p-Ser-FOXO3a expression were measured in caAKT transfected cells. α-Tubulin was used as a loading control. (B) Expression of Bim and p-FOXO3a were measured in vector- and caAKT-transfected cells after AZD6244 treatment. (C) Dose-response curves are shown for AZD6244 in vector-transfected and caAKT-transfected Calu-6 and H3122 cells. Cells were exposed to increasing concentrations of AZD6244 for 96 hours. Cell viability was determined by SRB. (D) Apoptosis induction by AZD6244. Cells were treated with 3 µM AZD6244 for 72 hours, and apoptosis was analyzed as described in Fig. 3C. Numbers represent percentages of apoptotic sub-G_1_–phase cells. Data represent one of three independent experiments with similar results. *Columns*, mean; *bar*, SD. *, *P*<0.05, compared with the control vector transfected cells. (E) TUNEL assay were conducted as described in Fig. 3D. The representative photographs of Calu-6 are shown.

Our previous study showed that high levels of p-AKT are associated with resistance to AZD6244 in lung cancer cells. Because AKT is known to regulate FOXO3a phosphorylation, we further investigated whether endogenous p-AKT affects FOXO3a and subsequently Bim expression. For this purpose, we transfected sensitive cell lines Calu-6 and H3122 with a retroviral vector expressing GFP-tagged constitutively active AKT (caAKT). Cells transfected with an empty vector were used as a control. After a brief period of cell selection with Geneticin, activity of AKT was verified in caAKT-transfected cells by p-GSK3α/β antibody (Fig. 5A). High levels of endogenous p-Thr32-FOXO3a and p-Ser253-FOXO3a were detected in caAKT-transfected cells (Fig. 5A).

We then measured Bim expression in caAKT-transfected cells after treatment with AZD6244. As shown in Fig. 5B, BimEL, BimL, and BimS expression was suppressed after AZD6244 treatment for 4, 8, and 24 hours. We also detected higher expression of p-FOXO3a in caAKT-transfected cells than in control vector–transfected cells in these two cell lines (Fig. 5B).

Parental, vector-transfected, and caAKT-transfected cells were treated with various doses of AZD6244, and cell viability was determined 96 hours after treatment. Results showed that transfection with caAKT made Calu-6 and H3122 cells resistant to AZD6244 (Fig. 5C). IC_50_ values to AZD6244 in parental or vector-transfected Calu-6 cells were 1.3 µM and 0.9 µM, respectively; the IC_50_ value in caAKT-transfected cells was 98.2 µM. The IC_50_ values for AZD6244 parental, vector-transfected, and caAKT-transfected H3122 cells were 2.4, 2.9, and 76.3 µM, respectively. Cell cycle analysis showed the inhibition of AZD6244-induced apoptosis in caAKT-transfected cells. In Calu-6 cells, the percentage of sub-G_1_ apoptotic cells decreased from 42% to 9.6% after treatment with AZD6244 for 72 hours. In H3122 cells, the percentage decreased from 36.9% to 10.4% (Fig. 5D). We also found that caAKT stable transfection suppressed AZD6244-induced apoptotic cells determined by TUNEL assay, from 48.5% to 15.9% in Calu-6 and 50.4% to 19.4% in H3122 cells (Fig. 5E).

## Discussion

In this study, we demonstrated that up-regulation of Bim is critical in ADZ6244-induced apoptosis. We also showed that the AKT/FOXO3a pathway is involved in the regulation of Bim expression induced by AZD6244. AZD6244 is a small-molecule inhibitor selective for MEK1/2. It has been investigated in clinical trials for the treatment of melanoma, advanced non–small cell lung cancer (NSCLC) and a variety of other malignancies. The mechanism by which this compound induces apoptosis has not been identified. The intrinsic apoptosis pathway, also known as the mitochondrial pathway, plays a critical role in chemotherapy and/or targeted therapy induced apoptosis. Recently, it has been shown that Bim is a key effector of tyrosine kinase inhibitor–induced apoptosis in human leukemia and melanoma cells [6]. Bim has also been shown to mediate epidermal growth factor receptor (EGFR) inhibitor–induced apoptosis in lung cancer cells that have EGFR or BRAF mutations [7], [8]. However, little is known about its role in regulating apoptosis in response to AZD6244 treatment.

In this study, we used a panel of lung cancer cell lines to identify apoptosis-resistance mechanisms that inhibit the activity of AZD6244 in lung cancer cells. First, our results showed that Bim is critical in apoptosis induced by the MEK inhibitor AZD6244. Second, FOXO3a, regulated by p-AKT and p-ERK, is a direct transcriptional regulator of Bim. Induction of apoptosis by the MEK inhibitor AZD6244 required a low level of endogenous p-FOXO3a (Thr32 and Ser253). Third, expression of constitutively active AKT could up-regulate p-FOXO3a (Thr32 and Ser253) and induce resistance to MEK inhibition.

We have previously shown that p-AKT expression is low in AZD6244-sensitive lung cancer cell lines but high in resistant cells, suggesting that p-AKT is a potential biomarker of sensitivity to AZD6244 treatment. Moreover, the down-regulation of p-AKT with transfected dominant-negative AKT sensitized resistant cells to AZD6244. In this study, we determined that AZD6244 treatment can strongly induce Bim expression in all three sensitive cell lines but not in resistant cells. Increased Bim levels in both protein and mRNA expression were detected with Western blotting and real-time PCR, respectively in sensitive cells. Knockdown of Bim with siRNA in the sensitive Calu-6 and H3122 cell lines increased the IC_50_ value to AZD6244 and substantially decreased apoptosis. This data clearly demonstrates that Bim is an important intermediary in AZD6244-induced apoptosis.

Both the Ras/Raf/MEK/ERK pathway and the PI3K/AKT pathway mediate signals from various growth factor receptors, and these two pathways regulate several common downstream molecules that are critical in cell survival and cell cycle progression such as forkhead transcription factors [21], cyclin D1 [22], Bad [23] and caspase-9 [24]. In our study, we determined endogenous expression levels of total FOXO3a, p-Thr32-FOXO3a, and p-Ser253-FOXO3a in all sensitive and resistant cell lines. Except for the sensitive H2347 cell line, which showed lower expression, the expression of total FOXO3a was not noticeably different between the sensitive and resistant cell lines. As we expected, basal levels of p-Thr32-FOXO3a and p-Ser253-FOXO3a were higher in resistant cells, which was consistent with higher levels of p-AKT expression shown in our previous study. Moreover, AZD6244 treatment did not alter the expression of p-Thr32-FOXO3a and p-Ser253-FOXO3a in any of the cell lines.

**Figure 6:**
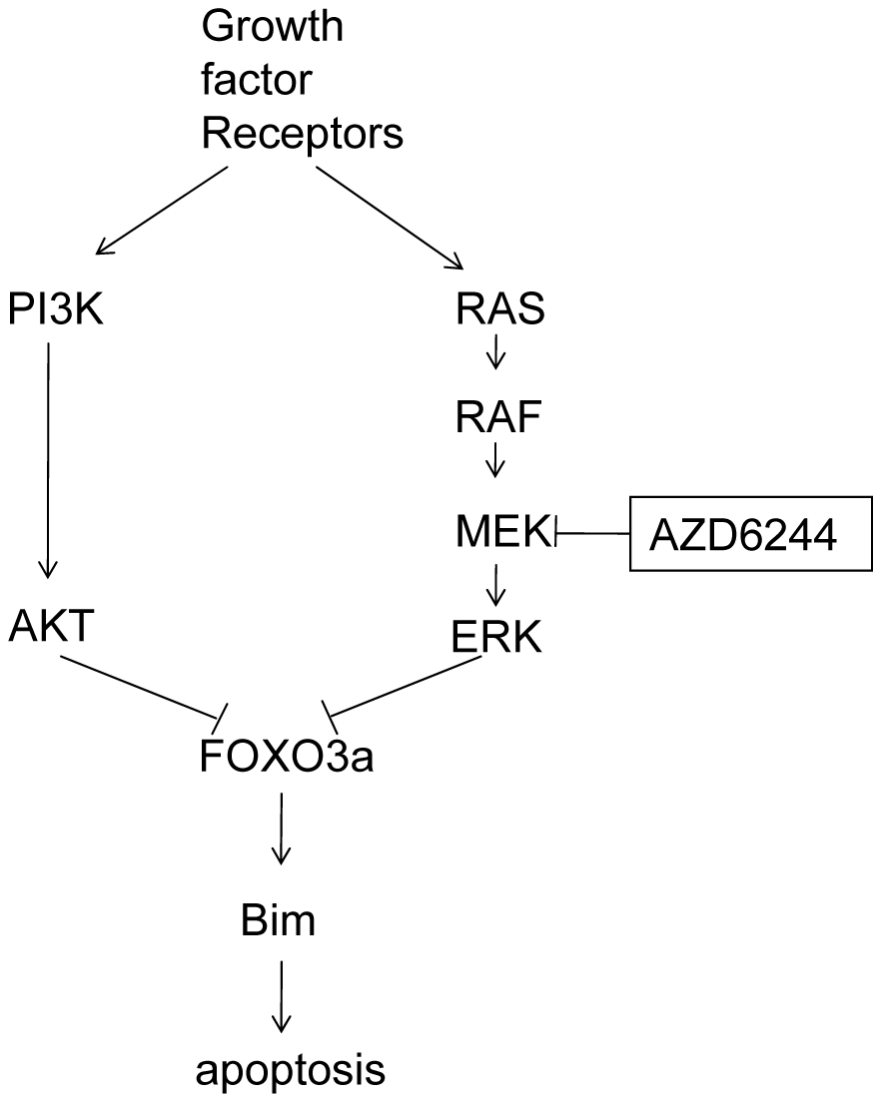
Model depicting the signaling pathways utilized by AZD6244 in lung cancer cells to induced Bim activation and subsequent apoptosis. Our results suggest that AZD6244-induced up-regulation of Bim is mediated by FOXO3a, which is regulated through both the MAPK/ERK and PI3K/AKT pathways. In sensitive cells, MEK inhibition is sufficient to induce expression of the downstream molecule, Bim, and to induce apoptosis. However, in resistant cells, in which the PI3K/AKT/FOXO3a pathway is constitutively activated, suppression of ERK is insufficient to induce apoptosis because of suppression of Bim expression.

We hypothesize that, in cells with high levels of p-Thr32-FOXO3a and p-Ser253-FOXO3a, the transcriptional function of FOXO3a was not activated after AZD6244 treatment because the down-regulated ERK could not suppress p-FOXO3a to a level sufficient to induce nuclear translocation of FOXO3a (Fig. 6). Our hypothesis was substantially supported with the immunofluorescence results, shown in Fig. 4B. We noted that in untreated Calu-6 cells with low p-FOXO3a expression, FOXO3a resided in both the cytoplasm and nucleus, whereas in untreated H522 cells, most of the FOXO3a resided in the cytoplasm and nuclear staining was negligible because phosphorylation retained the FOXO3a in the cytoplasm. After treatment with AZD6244, FOXO3a was dephosphorylated and activated, which ultimately explained the overall cellular response to AZD6244. In the sensitive cells, AZD6244-induced apoptosis was associated with FOXO3a dephosphorylation and nuclear translocation; in the resistant cells, however, dephosphorylation of FOXO3a at ERK sites was neutralized by a high level of endogenous p-FOXO3a at AKT sites which reduced expression of the target-molecule, Bim. We also determined that when FOXO3a was suppressed with a specific siRNA, the AZD6244-induced increase in Bim was strongly inhibited. These findings suggest that FOXO3a functions as a direct transcriptional regulator of Bim expression in lung cancer cell lines, which is consistent with previous reports in breast cancer [6], NSCLC [9], colon cancer [25] and leukemia [26].

It has been reported that a wide range of external stresses and stimuli, including DNA damage, microtubule disruption, or growth factor withdrawal, can induce overexpression of the proapoptotic BH3-only Bim, leading to apoptosis [27]. Accumulating evidences indicated that multiple mechanisms might contribute to Bim overexpression, including transcriptional up-regulation, protein phosphorylation or stabilization [28]. Our results showed that both transcriptional up-regulation and protein stabilization contributed to AZD6244-induced Bim accumulation in human lung cancer cells. Although how the two mechanisms interact and cooperate in Bim accumulation remains to be determined, our results also showed that the PI3K/AKT/FOXO3a pathway plays a critical role in the transcriptional regulation of Bim expression.

We and others have previously shown that constitutively active AKT was associated with resistance to chemotherapeutic and molecular-targeted drugs, including paclitaxel, AZD6244, tumor necrosis factor–related apoptosis-inducing ligand and cisplatin [5], [29]–[31]. To investigate how the constitutively activity AKT imparts resistant to AZD6244, we transfected caAKT into sensitive cell lines Calu-6 and H3122. Notably increased levels of p-Thr32-FOXO3a and p-Ser253-FOXO3a were detected in both cell lines after transfection. With AZD6244 treatment, Bim expression was inhibited in caAKT-transfected cells compared with control cells.

Cell death can be caused by different mechanisms, including apoptotic, autophagic and necrotic death. Apoptosis is an intracellular programmed cell death involving activation of the cysteine proteases (caspases) cascade [32]. The markers of apoptosis include cleavage of PARP1, release of cytochrome c from mitochondrial cleavage of chromosomal DNA, and activation of caspases [33], [34]; autophagic death involves a process of self-digestion of cellular material through formation of lysosome-like autophagosomes [35], [36]; and necrosis is a passive death process caused by external factors and involves loss of cellular homeostasis [37]. In this study, the western blot and TUNEL assay results showed that AZD6244 induced apoptosis after 4–48 h treatment. It is not clear if AZD6244 still induced cell apoptosis after 96 h treatment, although our anti-proliferation assay showed AZD6244 induced cell death after this treatment duration. It is possible that a relatively long-term treatment may cause an apoptosis-independent cell death or a mixture of apoptotic and non-apoptotic cell death.

In summary, our results indicated that FOXO3a is important to the antiproliferative effect of AZD6244 and induces mitochondrial apoptosis mediated by Bim. On the basis of our observations, we plan to focus on the PI3K/AKT/FOXO3a pathway and BH3-only proteins in the development of strategies to overcome resistance to AZD6244 in lung cancer cells.
